# Probabilistic risk assessment of dietary exposure to benzophenone derivatives in cereals in Taiwan

**DOI:** 10.1111/risa.14352

**Published:** 2024-06-22

**Authors:** Yu‐Fang Huang, Yun‐Ru Ju, Hsin‐Chang Chen

**Affiliations:** ^1^ Institute of Environmental and Occupational Health Sciences School of Medicine National Yang Ming Chiao Tung University Taipei Taiwan; ^2^ Department of Safety Health and Environmental Engineering National United University Miaoli Taiwan; ^3^ Department of Chemistry College of Science Tunghai University Taichung Taiwan

**Keywords:** Bayesian Markov‐chain Monte Carlo, benzophenone derivative, cereal, risks

## Abstract

Benzophenone (BP) and BP derivatives (BPDs) are widely used as ultraviolet (UV) stabilizers in food packaging materials and as photoinitiators in UV‐curable inks for printing on food‐contact materials. However, our knowledge regarding the sources and risks of dietary exposure to BP and BPDs in cereals remains limited, which prompted us to conduct this study. We measured the levels of BP and nine BPDs—BP‐1, BP‐2, BP‐3, BP‐8, 2‐hydroxybenzophenone, 4‐hydroxybenzophenone, 4‐methylbenzophenone (4‐MBP), methyl‐2‐benzoylbenzoate, and 4‐benzoylbiphenyl—in three types of cereals (rice flour, oatmeal, and cornflakes; 180 samples in total). A Bayesian Markov‐chain Monte Carlo (MC) simulation approach was used for deriving the posterior distributions of BP and BPD residues. This approach helped in addressing the uncertainty in probabilistic distribution for the sampled data under the detection limit. Through an MC simulation, we calculated the daily exposure levels of dietary BP and BPDs and corresponding health risks. The results revealed the ubiquitous presence of BP, BP‐3, and 4‐MBP in cereals. Older adults (aged >65 years) had the highest (97.5 percentile) lifetime carcinogenic risk for BP exposure through cereals (9.41 × 10^−7^), whereas children aged 0–3 years had the highest (97.5 percentile) hazard indices for BPD exposure through cereals (2.5 × 10^−2^). Nevertheless, across age groups, the lifetime carcinogenic risks of BP exposure through cereals were acceptable, and the hazard indices for BPD exposure through cereals were <1. Therefore, BPD exposure through cereals may not be a health concern for individuals in Taiwan.

## 1 INTRODUCTION

Benzophenone (BP) and BP derivatives (BPDs) are widely used as ultraviolet (UV) filters in personal care products, as UV stabilizers in food packaging materials, and as photoinitiators in UV‐curable inks used for printing on food‐contact materials (EFSA, 2009a; Suzuki et al., 2005). Humans are exposed to BPDs through inhalation, dermal contact, and consumption. In addition, exposure can occur through the consumption of BPD‐containing food‐flavoring agents, intake of BPD‐containing water or seafood, and migration from food packaging (IARC, 2013). Hazardous compounds such as BP and BPDs (e.g., 4‐methylbenzophenone [4‐MBP], methyl‐2‐benzoylbenzoate [M2BB], and 4‐benzoylbiphenyl [PBZ]) frequently contaminate food items through migration from their packaging materials (Jiménez‐Díaz et al., 2014). The risk of BPD contamination in food was first reported in 2009: the Rapid Alert System for Food and Feed in Europe announced that 4‐MBP can migrate (at a level of 798 ng/g) from cardboard packaging materials into cereal products. Subsequently, Belgian authorities reported that many breakfast cereals contained up to 4210 ng/g BP and 3729 ng/g 4‐MBP (EFSA, 2009b). However, these studies did not include dietary exposure assessments because of the unavailability of methods for the random sampling of food (EFSA, 2009b).

Safety concerns regarding BP have increased since the International Agency for Research on Cancer identified BP to be a potential carcinogen (Group 2B) on the basis of animal studies on its effects on the liver and kidney (IARC, 2013). In vitro and in vivo toxicological studies have demonstrated that BP and the BPDs BP‐1, BP‐2, BP‐3, BP‐8, 2‐hydroxybenzophenone (2‐OHBP), 4‐hydroxybenzophenone (4‐OHBP), and M2BB exert endocrine‐disrupting effects on Michigan Cancer Foundation‐7 (MCF‐7) human breast cancer cells and rats (Morizane et al., 2015; Nakagawa & Tayama, 2002; Suzuki et al., 2005). Because of their carcinogenic properties, endocrine toxicity, and increasing use, BPDs have drawn the attention of international regulators and agencies. The European Union (EU) Commission (1995), European Chemicals Agency (2020), EU Commission (Regulation No 10/2011; European Commission, 2011), and Council of Europe (2009) have set the daily oral reference dose (RfD) for BP, BP‐3, 2‐OHBP, 4‐OHBP, 4‐MBP, M2BB, and PBZ to 10−312 μg/kg/day. Because of the widespread exposure to BPDs in daily life, an assessment of BPD safety is essential; estimating exposure is a crucial step in characterizing its potential health risks.

Several studies have investigated the presence of BPDs in cereal‐based products and the migration of these BPDs into packaged food items (Bugey et al., 2013; Chang et al., 2019; Van Den Houwe et al., 2016). However, data regarding the sources of human exposure to BPDs in cereals remain limited. Being major sources of carbohydrates, protein, fiber, and trace minerals, cereal‐based products are staple foods for most populations (WHO, 2003). Because of the benefits of dietary fibers and whole grains, a diet high in these components may reduce the risks of type 2 diabetes, obesity, and cardiovascular disease (Aune et al., 2016; Ley et al., 2014; Seal & Brownlee, 2015). In the present study, to mitigate uncertainties in risk analysis, we adopted a probabilistic approach (Monte Carlo [MC] simulation) and thus realistically estimated health risks from BPD exposure through cereals. Because the levels of BP and BPDs in cereals may fall below the limit of detection (LOD), a Bayesian Markov‐chain MC (MCMC) approach was used to simulate the posterior distributions of chemical residues lower than the LOD (Kennedy, 2010; Lu et al., 2020). This assessment was performed in accordance with the risk assessment paradigm of the National Research Council (NRC, 1983)—an approach similar to that used by Lu et al. (2020) for detecting pesticides and estimating their occurrences in food items (Lu et al., 2020).

We first established the baseline residual levels of BP and nine BPDs—BP‐1, BP‐2, BP‐3, BP‐8, 2‐OHBP, 4‐OHBP, 4‐MBP, M2BB, and PBZ—in 180 cereals sampled considering their ingestion rates (IRs) in Taiwan—determined using data from the National Food Consumption Database of Taiwan (NFCDT). Second, we compared BP and BPD residue distributions evaluated using the MCMC and MC approaches. Finally, we measured the estimated daily intake (EDI) and cumulative health risks of BP and BPD exposure through cereals.

## 2 MATERIALS AND METHODS

### 2.1 Food sampling and analysis

Between January and June 2020, three batches of 180 breakfast cereal samples were purchased from supermarkets in Taiwan. The following three types of cereals were selected on the basis of data from the NFCDT: rice flour, oatmeal, and cornflakes. Samples with different packaging types (tin cans, laminated aluminum foil bags, and plastic bags) were selected. On the basis of the principle of sample proportionality and availability, 59, 61, and 60 samples of rice flour, oatmeal, and cornflakes, respectively, were randomly collected. Tin cans, laminated aluminum foil bags, and plastic bags were used to pack 7, 41, and 11 rice flour samples; 10, 39, and 12 oatmeal samples; and 0, 28, and 31 cornflake samples, respectively. All samples were collected shortly after the manufacture date. For each sample, the sampling date, manufacture date, packaging material, and raw material source (domestic or foreign) were recorded. The samples were preserved at room temperature and analyzed within 1 week from the date of sampling.

The qualification and quantification of BPDs in cereals were analyzed through ultrahigh‐performance liquid chromatography (UHPLC) coupled with tandem mass spectrometry (MS/MS) with isotope‐labeled internal standards (Huang et al., 2021; Liu et al., 2022). A homogenized cereal sample (0.5 g) was extracted using the fast pesticide extraction procedure and analyzed through UHPLC–MS/MS (LCMS−8045, Shimadzu). The MS/MS analysis was performed using a triple quadrupole with an electrospray ionization source. Ions were monitored in the positive and negative multiple reaction monitoring modes. In general, the LODs and limits of quantification were 0.001−0.289 and 0.003−0.867 ng/g for BP and BPDs, respectively. The mean recovery range and its intraday relative standard deviation (SD) were 79%−121% and 1.4%−20.8%, respectively. The analysis results were validated in accordance with the Codex Alimentarius Commission guidelines (Codex Alimentarius, 2017).

### 2.2 Risk assessment

**FIGURE 1:**
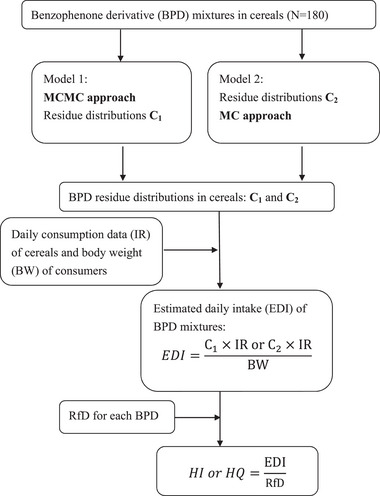
Flowchart of dietary exposure to benzophenone (BP) and BP derivatives (BPDs) mixtures in cereals. MC, Monte Carlo; MCMC, Markov‐chain MC.

Figure 1 presents a flowchart depicting dietary exposure to BP and BPD in cereals. Two modeling strategies were used to assess exposure. Models 1 and 2 were constructed using the MCMC (*C*_1_) and MC (*C*_2_) approaches, respectively, to derive the posterior distributions of BP and BPD residues. Simultaneously, BP and BPD residues were simulated using the MCMC and MC approaches to measure dietary exposure levels. Dietary BP and BPD exposure was estimated by dividing the daily cereal IR by consumer body weight (BW; exposure was calculated for various age groups; Section 2.2.1). Health risks were estimated by dividing the average daily intake by RfD (Sections 2.2.2 and 2.2.3).

#### 2.2.1 EDI values of BPDs in cereals

Dietary intake of BPDs through cereals was estimated through BPD residue simulation (*C*_1_ or *C*_2_; ng/g), using the IR (g/day) and mean BW (kg) for selected age groups. The formula used was as follows:

(1) $$\mathrm{EDI}=\left(C_{1}\times \mathrm{IR}\mathrm{or}C_{2}\times \mathrm{IR}\right)/\mathrm{BW}\left(\mathrm{ng}/\left[\mathrm{kg}\;\mathrm{day}\right]\right)$$

The mean cereal IRs (g/day) and BWs (kg) of seven age groups (0−3, 3−6, 6−12, 12−16, 16−18, 19−65, and >65 years) were used to estimate the daily dietary intake of BPDs. These data were obtained from the NFCDT (TFDA, 2019) and are presented in Table S1.

#### 2.2.2 Estimation of noncarcinogenic risk

For the seven age groups, noncarcinogenic risk from BPD exposure was estimated using a hazard quotient (HQ) approach, in which EDI was divided by RfD. According to the EU Commission (Regulation No 10/2011; European Commission, 2011) and Council of Europe (2009) report, the RfD values of BP, 4‐MBP, and BP‐3 are 30, 30, and 100 μg/kg/day, respectively. The hazard index (HI) was used to assess the cumulative exposure to BPDs; this parameter was calculated by summing the HQs of all BPDs. BP and BP‐3 exert endocrine‐disrupting effects on the human breast cancer cell line MCF‐7 and rats. Notably, 4‐MBP and BP have similar chemical structures. BP, 4‐MBP, and BP‐3 exhibit identical modes of action. Therefore, the probabilistic HI distribution under the assumption of additive effects on analyte interactions was estimated as follows: HI = ∑*HQ*_BPDs_ = HQ_BP_ + HQ_4‐MBP_ + HQ_BP‐3._ Seven BPDs were excluded because of their low detection frequencies (DFs). Noncarcinogenic risks were estimated on the basis of two BPD residue (*C*_1_ and *C*_2_) simulations. An HQ or HI of <1 indicated that noncarcinogenic risk from cereal consumption was almost negligible for Taiwanese people in the corresponding age group.

A sensitivity analysis was performed to estimate the contribution of different variables (e.g., BP, 4‐MBP, and BP‐3 levels; IRs of in rice flour, oatmeal, and corn flakes; and BWs) to the BPD HIs obtained through the MCMC and MC approaches. The analysis was performed for the age group with the highest HI: children aged 0−3 years, who have low BW but relatively high cereal intake. However, because milk is the main nutrient source for children aged 0−3 years, preschool children (aged 3−6 years), and older adults, groups with the second highest HI (estimated through MCMC and MC methods) were selected for the sensitivity analysis.

#### 2.2.3 Estimation of carcinogenic risk

BP is a potential renal carcinogen; its mode of action involves renal degeneration. The effect of BP exposure may have a linear relationship with BP dose (US EPA, 2005). Carcinogenic risk from BP exposure through cereals as estimated in terms of lifetime cancer risk (LTCR): LTCR = CDI × CSF (US Environmental Protection Agency [EPA], 2005), where CDI is the chronic daily intake of BP (mg/kg/day), and CSF is the cancer slope factor for BP [4.8 × 10^−3^ (mg/kg/day)^−1^] (Michigan Department of Environmental Quality, 2015). CDI was estimated using EDI, exposure duration (ED; 2, 6, 12, 16, 18, 65, and 70 years for age groups 0−3, 3−6, 6−12, 12−16, 16−18, 19−65, and >65 years), exposure frequency (EF; 365 days/year of age), and average exposure time (AT = average life span of 70 years × 365 days/year = 25,550 days): CDI = (EDI × ED × EF)/AT. The mean (97.5th percentile [P97.5]) EDI (obtained using the MCMC approach) for the seven age groups was used to calculate LTCR. For instance, for the* >*65‐year age group, the P97.5 LTCR was calculated as follows: (1.96 × 10^−4^ mg/kg/day × 70 years × 365 days/year)/(25,550 days) × 4.8 × 10^−3^ (mg/kg/day) ^−1^.

### 2.3 Statistical analysis

#### 2.3.1 MCMC approach

Of the eight BPDs detected in this study, only BP, 4‐MBP, and BP‐3 were detected in sufficient numbers of samples (i.e., DFs > 59%). Using the MCMC approach, we predicted the distributions of BP, 4‐MBP, and BP‐3 residues, including those higher and lower than the LODs, thus addressing uncertainty associated with other established methods (Kennedy, 2010; Lu et al., 2020; Paulo et al., 2005). The BPD residue distribution (*C*_1_) was log–normal; thus, the natural logarithm of BPD residues was modeled as a normal distribution: ln(*C*_1_) ∼ *N*(*μ*, *σ* ^2^), where μ and σ denote the mean and SD of the posterior distribution, respectively. To estimate the probability of residues lower than the LODs being present, the number of samples with BPDs lower than the LOD were considered to follow a binomial distribution (*N*_0_):

(2) $$N_{0}∼\mathrm{Bin}\left(N,p_{0}+\left(1-p_{0}\right)\Phi \left(a\right)\right),$$

where *N* is the total number of samples with BPDs, *p*_0_ is the probability of a batch having 0 residues, *α* is (ln (LOD) − μ)/σ, and Φ(*α*) is the cumulative standard normal distribution of *α*. The parameters of the prior distribution, namely, *p*_0,_ μ, and σ^−2^, were assumed to be *p*_0_∼*β*(1, 1), μ∼*N*(0, 0.01), and σ^−2^∼*γ*(0.1, 0.1) in the respective distribution in the MCMC model (Paulo et al., 2005).

To estimate the mean levels of BP and BPDs in cereals from their posterior distributions, we performed an MCMC simulation for the posterior mean as follows:

(3) $$E\left(C_{1}\right)=\left(1-p_{0}\right)\exp\left(\mu +\sigma^{2}/2\right),$$

where *C*_1_ is the mean of the posterior distribution in cereals (ng/g). The MCMC simulation was performed using WinBUGS (Spiegelhalter et al., 1996). Approximately 14,000 samples were drawn from the posterior distribution; the first 4000 samples were discarded to create a burn‐in period.

#### 2.3.2 MC approach

A probabilistic model (i.e., an MC simulation; Crystal Ball, version 11.1.2.4; Oracle) was used to characterize parameter variability so as to estimate dietary intake and associated noncarcinogenic and carcinogenic risks. For MC simulations, the BP and BPD residues (*C*_2_) and IRs of the cereals were distributed log–normally, and the BWs for each age group were normally distributed. Random sampling was performed, with a simulation of 10,000 iterations, to obtain the EDI and HI (95% confidence interval) for each age group. The P97.5 cutoffs, the high exposure scenario, were used to estimate the EDI and noncarcinogenic and carcinogenic risks for each age group.

The BPD residue levels are presented in terms of the mean (SD) values and ranges. The correlations of BP and BPDs levels in cereals with each cereal's source (domestic or foreign) were assessed using Spearman correlation coefficients. One‐way analysis of variance (ANOVA) was used to compare the BPD residues across cereals and packaging materials. The Tukey test was used for post hoc comparisons. Statistical analysis was performed in SPSS (version 19.0; SPSS), and the level of significance was set at *p* < 0.05.

## 3 RESULTS AND DISCUSSION

### 3.1 BPD residues in cereals

Table 1 lists the DFs and distributions of BP and BPDs in the rice flour, oatmeal, and cornflakes samples. In the cereals, BP and two BPDs (4‐MBP and BP‐3) were detected at DFs of >59%, five BPDs (BP‐1, BP‐2, 4‐OHBP, M2BB, and PBZ) were detected at DFs of 2%−17%; and two BPDs (BP‐8 and 2‐OHBP) were detected at DFs of lower than the LOD. BP was detected in all samples, and 4‐MBP and BP‐3 exhibited sufficient DFs in 95% and 63% of the samples, respectively. These results indicate that BP, 4‐MBP, and BP‐3 were present in all three types of cereals. BP and 4‐MB are the most widely used photoinitiators in printing ink in several European countries (Bugey et al., 2013; EFSA, 2009a; Gallart‐Ayala et al., 2011; Jung et al., 2013).

**TABLE 1:** Summary of the detection frequencies (DFs) and distribution profiles of benzophenone (BP) derivatives (BPDs) in cereals (*N* = 180) unit: ng/g.

Cereal types		BP	4‐MBP	BP‐3	BP‐1	BP‐2	BP‐8	2‐OHBP	4‐OHBP	M2BB	PBZ
Rice flour (*n* = 59)	DF (%)	100	85	59	0	0	0	0	5	0	2
	Mean (SD)	37.64 (19.65)	1.80 (1.58)	0.37 (0.32)	<0.18	<0.02	<0.03	<0.32	17.01 (14.97)	<0.39	0.94 (‐)
	Range	16.79–108.93	<0.01–11.85	<0.06–0.86	<0.18	<0.02	<0.03	<0.32	<0.51–33.26	<0.39	<0.07–0.94
Oatmeal (*n* = 61)	DF (%)	100	100	69	2	2	0	0	0	0	2
	Mean (SD)	21.04 (8.44)	4.21 (8.29)	0.24 (0.21)	1.21 (‐)	0.16(‐)	<0.08	<0.14	<0.04	<0.28	0.42 (‐)
	Range	13.57–67.51	1.21–65.8	<0.05–1.02	<0.04–1.21	<0.02–0.16	<0.08	<0.14	<0.04	<0.28	<0.05–0.42
Cornflakes (*n* = 60)	DF (%)	100	100	62	0	0	0	0	17	8	0
	Mean (SD)	89.14 (198.71)	2.59 (1.78)	1.18 (1.26)	<0.043	<0.02	<0.07	<0.15	4.89 (2.24)	5.34 (6.88)	<0.05
	Range	22.35–1083.84	0.9–12.04	<0.07–8.53	<0.04	<0.02	<0.07	<0.15	<0.05–9.66	<0.29–17.33	<0.05
Total cereals (*N* = 180)	DF (%)	100	95	63	1	1	0	0	7	3	1
	Mean (SD)	49.18 (118.37)	3.03 (5.18)	0.66 (0.82)	1.21 (‐)	0.16 (‐)	<0.03	<0.14	7.69 (8.33)	5.34 (6.88)	0.68 (0.37)
	Range	13.57–1083.84	<0.01–65.8	<0.05–8.53	<0.04–1.21	<0.02–0.16	<0.03	<0.14	<0.04–33.26	<0.28‐17.33	<0.05–0.94

Abbreviations: 2‐OHBP, 2‐hydroxybenzophenone; 4‐OHBP, 4‐hydroxybenzophenone; M2BB, methyl‐2‐benzoylbenzoate; 4‐MBP, 4‐methylbenzophenone; PBZ, 4‐benzoylbiphenyl; SD, standard deviation.

Data on the levels of BP and BPD in cereals are limited. BP was the main contributor to the total BP and BPD residues, with the level being the highest (mean [SD, range]) in cornflakes (89.1 [198.7, 22.4−1083.8] ng/g), followed by rice flour (37.6 [19.7, 16.8−108.9] ng/g) and oatmeal (21.0 [8.4, 13.6−67.5] ng/g; *p* < 0.05, one‐way ANOVA). The level of BP residue estimated in the present study was lower than those reported in Swiss (Bugey et al., 2013) and German (Jung et al., 2013) studies (5 −7 × 10^6^ and 3367–3413 ng/g, respectively) but higher than those reported in Belgian (Van Den Houwe et al., 2016) and Spanish (Gallart‐Ayala et al., 2011) studies (nondetectable to 20 and nondetectable to 40 ng/g, respectively). The second highest contributor to BPD residue was 4‐MBP, at the mean (SD, range) concentrations of 1.8 (1.6, 0.01−11.9), 4.2 (8.3, 1.2−65.8), and 2.6 (1.8, 0.9−12.0) ng/g in rice flour, oatmeal, and cornflakes, respectively (*p* > 0.05). These values were lower than those reported in Belgium (795–5400 ng/g; EFSA, 2009a) and Germany (65–8073 ng/g; Jung et al., 2013). BP‐3 exhibited relatively low mean (SD, range) residual concentrations: 0.4 (0.3, 0.1−0.9), 0.2 (0.2, 0.1−1.0), and 0.8 (1.1, 0.1−8.5) ng/g in rice flour, oatmeal, and cornflakes, respectively (*p* < 0.05). The mean residual concentrations of the other BPDs with low (<17%) DFs were 0.2−1.2 ng/g, with the exception of 4‐OHBP and M2BB, which exhibited concentrations of 7.7 and 5.3 ng/g, respectively.

The Spearman correlations among the residual BP, 4‐MBP, and BP‐3 in the cereal samples were determined (Table S2). A strong correlation was found between BP and BP‐3 (*r* = 0.74, *p* < 0.001), and moderate correlations were noted between BP and 4‐MBP (*r* = 0.38, *p* < 0.001) and between 4‐MBP and BP‐3 (*r* = 0.34, *p* < 0.001). These correlations among BP and BPDs in cereals indicate that they are from similar sources.

In cereals, the potential sources of BP and BPD residues are the flavoring ingredients and the migration of these compounds from food packaging materials. When the findings were stratified by packaging materials, the cereal samples packed in plastic had the highest mean BP levels (95.6 ng/g), followed by samples packed in aluminum foil bag (29.6 ng/g) and tin cans (26.9 ng/g; *p* = 0.002, one‐way ANOVA). Cereal samples packed in plastic also had significantly higher mean BP‐3 levels (1.1 ng/g) than did those packed in aluminum foil bags (0.6 ng/g) and tin cans (0.3 ng/g; *p* = 0.01, one‐way ANOVA). No significant differences were noted among packaging materials in the mean 4‐MBP level. Foreign sources of raw packaging materials exhibited weak but significant correlations with BP (*r* = 0.20, *p* = 0.01) and BP‐3 (*r* = 0.44, *p* < 0.001). Considerable levels of BP (1084 and 1055 ng/g) were detected in two muesli samples packed in a polylactide material sourced from Germany. Although no packaging materials were analyzed, we found that the presence of a BP residue may be related to the packaging material and its source (foreign or domestic). This finding is consistent with those of a report associating high levels of BP in food with the packaging materials (EFSA, 2009a).

### 3.2 MCMC modeling of BPDs

Table 2 presents the MCMC‐simulated distributions of BP, 4‐MBP, and BP‐3 residues. The means (SD) posterior log–normal distributions of BP, 4‐MBP, and BP‐3 were 36.78 (2.20), 1.74 (0.14), and 0.37 (0.04) ng/g in rice flour; 20.64 (0.91), 3.65 (0.42), and 0.19 (0.05) ng/g in oatmeal; and 65.28 (8.53), 2.50 (0.16), and 0.68 (0.08) ng/g in cornflakes, respectively. In all three types of cereals, the proportions of BP lower than the LOD were all 1.6%. These results confirmed the ubiquitous presence of BP in the cereal samples. The proportions of 4‐MBP and BP‐3, which were also lower than the LOD, were 16.3% and 40.1% in rice flour, 1.6% and 6.8% in oatmeal, and 1.6% and 38.7% in cornflakes, respectively.

**TABLE 2:** Posterior mean and standard deviations for parameters of the BPDs residue distributions.

Cereal types	*n*	Parameters	Compounds (ng/g)		
			BP	4‐MBP	BP‐3
Rice flour	59	*p*_0_ (%)	1.6 ± 1.6	16.3 ± 4.7	40.1 ± 6.2
		μ	3.53 ± 0.05	0.66 ± 0.05	−0.49 ± 0.03
		σ	0.42 ± 0.04	0.38 ± 0.04	0.18 ± 0.02
		Distribution	LN (36.78, 2.20)	LN (1.74, 0.14)	LN (0.37, 0.04)
Oatmeal	61	*p*_0_ (%)	1.6 ± 1.6	1.6 ± 1.6	6.8 ± 6.3
		μ	2.99 ± 0.04	1.9 ± 0.10	−2.51 ± 0.28
		σ	0.31 ± 0.02	0.75 ± 0.06	0.18 ± 0.02
		Distribution	LN (20.64, 0.91)	LN (3.65, 0.42)	LN (0.19, 0.05)
Cornflakes	60	*p*_0_ (%)	1.6 ± 1.5	1.6 ± 1.6	38.7 ± 6.1
		*μ*	3.84 ± 0.11	0.82 ± 0.06	0.01 ± 0.07
		*σ*	0.83 ± 0.08	0.45 ± 0.04	0.41 ± 0.05
		Distribution	LN(65.28, 8.53)	LN (2.50, 0.16)	LN (0.68, 0.08)
Total cereals	180	*p*_0_ (%)	0.5 ± 0.5	5.5 ± 1.7	2.4 ± 2.4
		*μ*	3.45 ± 0.05	0.85 ± 0.04	−2.62 ± 0.25
		*σ*	0.66 ± 0.03	0.57 ± 0.03	2.13 ± 0.18
		Distribution	LN (39.09, 2.15)	LN (2.60, 0.13)	LN (0.74, 0.26)

Figure S1 presents the mean (SD) values of BPD residues estimated using the MCMC and MC approaches for rice flour, oatmeal, and cornflakes. A comparison of the two approaches revealed similar mean values. However, the MC approach yielded higher SDs, particularly for BP in cornflakes. The MC approach can reduce uncertainties in exposure assessment (Huang et al., 2014; Lu et al., 2020). These simulation results were used to validate the mean (SD) levels of BP and 4‐MBP residues in the cereal samples with high detection rates.

### 3.3 EDI

**FIGURE 2:**
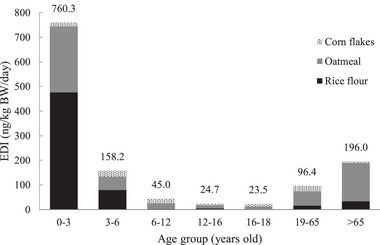
The P97.5 values of the dietary intake were estimated using the Markov‐chain Monte Carlo (MCMC) approach for three types of cereals and each age group. EDI, estimated daily intake.

The P97.5 values of dietary intake (estimated using the MCMC approach) for the three types of cereals and for each age group are presented in Figure 2. For all cereals, the P97.5 of EDI was the highest in the 0−3‐year age group (760.3 ng/kg/day), followed by the >65‐year age group (196.0 ng/kg/day) and the 3−6‐year age group (158.2 ng/kg/day). The highest contributors to the EDI of BPDs were rice flour in the 0−3‐year age group (450.7 ng/kg/day), oatmeal in the 0−3‐year age group (225.0 ng/kg/day), and rice flour in the 3−6‐year age group (75.1 ng/kg/day; Table S3).

Figure S2 presents the P97.5 EDI estimated using an MC simulation for each cereal and age group. The P97.5 EDI was the highest in the 0−3‐year age group (1017.1 ng/kg/day), followed by the >65‐year age group (200.2 ng/kg/day) and the 3−6‐year age group (181.7 ng/kg/day). BP was the primary contributor to EDI in the 0−3‐year age group, with its level being the highest in rice flour (476.7 ng/kg/day), followed by oatmeal (270.2 ng/kg/day) and cornflakes (140.5 ng/kg/day; Table S4). EDI values obtained using the MC approach were higher than those obtained using the MCMC approach; these findings indicate the relatively conservative nature of MCMC simulation. The EDI values estimated in this study are partially consistent with the results obtained by Cunha et al. (2018), who reported that weekly dietary BP‐3 exposure from seafood was 52 ng/kg—a value substantially lower than the tolerable weekly intake of 14,000 μg/kg (Cunha et al., 2018). Because of the high EDI of BP and BPD from cereal consumption and the role of BP in endocrine disruption, BP and BPD exposure in children aged 0−3 years warrants further research.

### 3.4 Risk from cereal consumption

**FIGURE 3:**
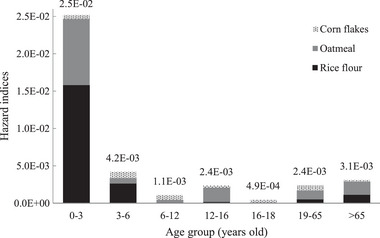
The P97.5 values of hazard indices (HIs) using the MCMC approach for three types of cereals and for each age group.

Few studies have analyzed the risk of BP exposure through cereal consumption. The highest P97.5 HIs obtained using the MCMC approach were 2.5 × 10^−2^, 4.2 × 10^−3^, and 3.1 × 10^−3^ in the 0−3‐, 3−6‐, and >65‐year age groups, respectively (Figure 3). Table S5 presents the mean (P97.5) HQs for BPDs (obtained using the MCMC approach) stratified by cereal type and age group. In the 0−3‐year age group, the P97.5 HQs for BP posed the highest risk for rice flour (1.5 × 10^−2^), followed by oatmeal (7.5 × 10^−3^). In the MC simulation, the highest P97.5 HIs were 2.9 × 10^−2^, 4.6 × 10^−3^, and 8.4 × 10^−3^ in the 0−3‐, >65‐, and 3−6‐year age groups, respectively (Figure S3). In the 0−3‐year age group, the P97.5 HQs for BP posed the highest risk for rice flour (1.6 × 10^−2^), followed by oatmeal (5.1 × 10^−3^) and cornflakes (4.7 × 10^−3^; Table S6). Table 3 lists the carcinogenic risks, indicated using MCMC‐based mean (P97.5) LTCRs, of BP exposure through cereals in the seven age groups. Many Chinese breakfast options are available in Taiwanese markets. However, we considered the worst exposure scenario—an EF of 365 days per year. These risks were within acceptable limits (10^−4^ to 10^−6^), according to the specifications of the US EPA (2011). The results revealed no carcinogenic risk from BP exposure through cereals; however, older adults (>65 years) had a slightly higher risk than individuals in other age groups. This finding corroborates that of a European study reporting no risk from consuming seafood containing BP‐1 and BP‐3 (Cunha et al., 2018).

**TABLE 3:** The mean (P97.5) lifetime cancer risk (LTCR) using the MCMC simulation of BP ingestion through cereal consumption for the seven age groups.

	Age groups
	0−3	3−6	6−12	12−16	16−18	19−65	>65
Mean (P97.5)	1.16 × 10^−4^	2.43 × 10^−5^	7.0 × 10^−6^	3.4 × 10^−6^	3.5 × 10^−6^	1.42 × 10^−5^	3.09 × 10^−5^
EDI (mg/kg/day)	(7.60 × 10^−4^)	(1.56 × 10^−4^)	(4.5 × 10^−5^)	(2.47 × 10^−5^)	(2.35 × 10^−5^)	(9.64 × 10^−5^)	(1.96 × 10^−4^)
ED (year)	2	6	12	16	18	65	70
EF (days/years)	365	365	365	365	365	365	365
AT (day)	25,550	25,550	25,550	25,550	25,550	25,550	25,550
Mean (P97.5)	1.59 × 10^−8^	9.99 × 10^−9^	5.76 × 10^−9^	3.73 × 10^−9^	4.32 × 10^−9^	6.33 × 10^−8^	1.48 × 10^−7^
LTCR	(1.04 × 10^−7^)	(6.42 × 10^−8^)	(3.70 × 10^−8^)	(2.71 × 10^−8^)	(2.90 × 10^−8^)	(4.29 × 10^−7^)	(**9.41 × 10^−7^**)

Abbreviations: AT, average exposure time; ED, exposure duration; EDI, estimated daily intake; EF, exposure frequency.

**FIGURE 4:**
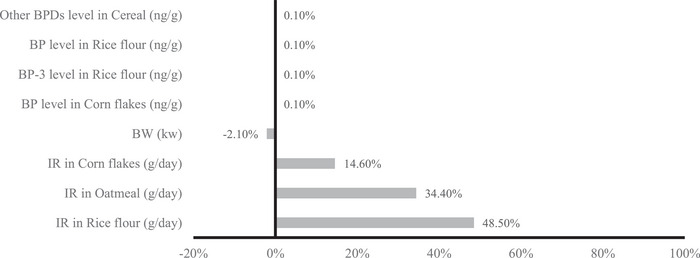
Sensitivity analysis of each parameter contributing to HI of BPDs using the MCMC method for children aged 3−6 years.

Sensitivity analyses performed for assessing the variables’ contributions to the BPD HIs (obtained using the MCMC approach) revealed that in children aged 3−6 years, the main contributors to HIs were the amounts of rice flour (48.5%) and oatmeal (34.4%) consumed (Figure 4). A similar was obtained using the MC approach: among older adults (age >65 years), the highest sensitivity was attributed to the amounts of oatmeal (65.7%) and rice flour (12.6%) consumed and the levels of BP in oatmeal (5.1%; Figure S4).

In exposure assessments, uncertainty can arise from the model inputs, exposure models, or insufficient understanding of exposure scenarios (IPCS, 2008). For dietary exposure to BP and BPDs, uncertainty may arise from the amounts of food consumed or the levels of residues in food. The uncertainty associated with residues in food was minimized using the UHPLC–MS/MS system. However, food consumption data were collected using a 24‐h dietary recall questionnaire, which might have introduced recall bias. Notably, uncertainty may also arise from insufficient data regarding toxicological mechanisms, modes of action, and correlations of modes of action with humans.

This study has some limitations. Several food items (e.g., seafood, milk, or other foods and beverages) were not included in this study. Therefore, the actual exposure levels might have been underestimated in this study. Furthermore, nondietary sources (e.g., personal care products) of BP and BPD exposure were not considered. A Greek study analyzing human biomonitoring data revealed that the average EDI_urine_ from BP‐type UV filters, calculated using a urinary level of 22.3 ng/mL, was 28.9 μg/kg/day (Asimakopoulos et al., 2014). This value was higher than the EDI in our study, possibly because the Greek study included only a small number of urine samples (*n* = 100). As such, both dietary and nondietary sources must be considered in the analysis of BPD exposure. In the future, studies should evaluate the biomarkers of BP and BPDs in urine as indicators of total exposure via different routes. In addition, studies are warranted to estimate the daily intake of BP and BPDs by using excretion fractions derived from a physiological pharmacokinetic model. Despite the aforementioned limitations, our study appears to be the first to use both MCMC and MC approaches to offer insights into the risk from BPDs exposure through cereals.

## 4 CONCLUSION

Our findings revealed the ubiquitous presence of BP, BP‐3, and 4‐MBP in cereals. The results of the MCMC simulation validated the estimated mean (SD) values of BP and 4‐MBP residues at a high detection rate. The P97.5 LTCR of BP (<9.41 × 10^−7^) was acceptable, and the HIs for BPDs exposure (<0.025) were <1 for all age groups, indicating that BPDs exposure through cereals is not a health concern for individuals in Taiwan. Although the carcinogenic risk from BP exposure was noted to be within an acceptable limit, a comprehensive survey should be conducted to analyze BPDs exposure through other food products, such as rice and milk, and personal care products.

## CONFLICT OF INTEREST STATEMENT

The authors declare no competing interest.

## Supporting information

Supplementary Material
