# Supplementary material for: Probabilistic risk assessment of dietary exposure to benzophenone derivatives in cereals in Taiwan
Source: Risk Anal. 2024 Jun 22;45(1):5–13. doi: 10.1111/risa.14352 (PMC11735342; doi:10.1111/risa.14352)
Supplement: Supplementary file 1 — Supplementary Material [file RISA-45-5-s001.docx]

**Supplementary material**

**Probabilistic risk assessment of dietary exposure to benzophenone derivatives in cereals in Taiwan**

**Yu-Fang Huang^1*^, Yun-Ru Ju^2^, Hsin-Chang Chen^3^**

^1^ Institute of Environmental and Occupational Health Sciences, School of Medicine, National Yang Ming Chiao Tung University, Taipei, Taiwan

^2^ Department of Safety, Health and Environmental Engineering, National United University, Miaoli, Taiwan

^3^Department of Chemistry, College of Science, Tunghai University, Taichung, Taiwan

^*^ Address correspondence to Yu-Fang Huang, School of Medicine, National Yang Ming Chiao Tung University, Taipei, Taiwan; Tel: 886-2-28267378; yfh@nycu.edu.tw

Pages:9; Tables:6; Figures:4.

Table S1 Risk assessment of cereal consumption for different age populations in Taiwan

Table S2 The Spearman correlations among the residual 4-MBP, and BP-3 in cereal samples

Table S3 The mean (P97.5) values of EDI for BP and BPDs using the MCMC approach for three types of cereals and for each age group

Table S4 The mean (P97.5) values of EDI for BP and BPDs using the MC approach for three types of cereals and for each age group

Table S5 The mean (P97.5) HQs for BP and BPDs using the MCMC method by cereal type and age group

Table S6 The mean (P97.5) HQs for BP and BPDs using the MC method by cereal type and age group

Figure S1. Comparison of means (SDs) of BP, 4-MBP, and BP-3 distributions using the MCMC and MC methods for rice flour, oatmeal, and cornflakes

Figure S2. The P97.5 values of the dietary intake estimated using the MC approach by cereal type and age group

Figure S3. The P97.5 values of the HIs using MC simulation by cereal type and age group

Figure S4. Sensitivity analysis of each parameter’s contribution to the BPDs HIs using the MC method for older adults aged >65 years

Table S1 Risk assessment of cereal consumption for different age populations in Taiwan

|  | |  | Age group (years old) | | | | | | | | | |
| --- | --- | --- | --- | --- | --- | --- | --- | --- | --- | --- | --- | --- |
|  | |  | 0－3 | | 3－6 | 6－12 | 12－16 | | 16－18 | 19－65 | | >65 |
| Intake rate, mean (SD) (g/day) | | | | | | | | | | | | |
| Rice flour | |  | 20.47 (130.44) | | 5.50(44.98) | 0.35 (8.38) | 1.06 (17.59) | | 0.27 (3.06) | 3.61 (36.57) | | 7.46 (39.88) |
| Oatmeal | |  | 20.45 (104.65) | | 6.22(56.18) | 4.56 (34.54) | 4.08 (34.54) | | 5.14 (40.26) | 21.81 (89.48) | | 61.95 (140.44) |
| Corn flakes | |  | 0.68 (12.12) | | 1.09(10.38) | 1.11 (14.72) | 0.92 (17.00) | | 1.58 (19.00) | 2.29 (33.00) | | 1.0 (17.13) |
| Body weight,  mean (SD) (kg) | | | 12.85 (3.01) | | 19.93 (4.05) | 37.11 (14.17) | | 55.00 (10.66) | 62.4 0(17.53) | | 63.18 (12.51) | 61.53 (10.36) |
|  | |  | | |  |  |  |  |  |  |  |  |

Table S2 The Spearman correlations among the residual 4-MBP, and BP-3 in cereal samples

|  | BP | BP-3 | 4-MBP | Foreign sources^a^ |
| --- | --- | --- | --- | --- |
| BP | 1 |  |  |  |
| BP-3 | 0.74* | 1 |  |  |
| 4-MBP | 0.38** | 0.34** | 1 |  |
| Foreign source^a^ | 0.20* | 0.44** | -0.10 | 1 |

^a^raw materials obtained from foreign sources; *p < 0.05 **p < 0.001

Table S3 The mean (P97.5) values of EDI for BP and BPDs using the MCMC approach for three types of cereals and each age group

| EDI (ng/kg/day) | BPDs | Age group (years old) | | | | | | |
| --- | --- | --- | --- | --- | --- | --- | --- | --- |
|  |  | **0-3** | **3-6** | **6-12** | **12-16** | **16-18** | **19-65** | **>65** |
| Rice flour,  Mean (P97.5) | BP | 74.47 | 10.29 | 0.37 | 0.76 | 0.16 | 2.11 | 4.63 |
|  |  | (450.65) | (75.12) | (2.48) | (4.89) | (1.05) | (14.86) | (32.14) |
|  | 4-MBP | 3.56 | 0.49 | 0.02 | 0.04 | 0.01 | 0.10 | 0.22 |
|  |  | (21.99) | (3.62) | (0.12) | (0.23) | (0.05) | (0.69) | (1.49) |
|  | BP-3 | 0.74 | 0.10 | 0.004 | 0.01 | 0.001 | 0.02 | 0.05 |
|  |  | (4.42) | (0.79) | (0.03) | (0.05) | (0.01) | (0.15) | (0.31) |
|  | ΣBPDs | 78.77 | 10.88 | 0.39 | 0.8 | 0.17 | 2.23 | 4.89 |
|  |  | (477.07) | (79.53) | (2.62) | (5.17) | (1.11) | (15.71) | (33.94) |
| Oatmeal,  Mean (P97.5) | BP | 29.34 | 8.54 | 3.15 | 1.51 | 1.62 | 6.9 | 21 |
|  |  | (224.95) | (45.53) | (20.06) | (10.21) | (10.44) | (49.97) | (131.32) |
|  | 4-MBP | 5.24 | 1.55 | 0.58 | 0.26 | 0.29 | 1.19 | 3.74 |
|  |  | (39.91) | (8.43) | (3.60) | (1.77) | (1.88) | (8.82) | (23.40) |
|  | BP-3 | 0.26 | 0.08 | 0.03 | 0.01 | 0.02 | 0.06 | 0.20 |
|  |  | (2.15) | (0.44) | (0.18) | (0.11) | (0.10) | (0.40) | (1.36) |
|  | ΣBPDs | 34.84 | 10.17 | 3.76 | 1.79 | 1.92 | 8.16 | 24.93 |
|  |  | (267.01) | (54.41) | (23.84) | (12.08) | (12.42) | (59.18) | (156.08) |
| Corn flakes,  Mean (P97.5) | BP | 2.01 | 3.13 | 2.74 | 0.75 | 1.31 | 3.60 | 1.06 |
|  |  | (15.55) | (23.12) | (17.61) | (7.15) | (9.52) | (20.45) | (5.71) |
|  | 4-MBP | 0.08 | 0.12 | 0.10 | 0.03 | 0.05 | 0.14 | 0.04 |
|  |  | (0.56) | (0.91) | (0.70) | (0.28) | (0.37) | (0.81) | (0.25) |
|  | BP-3 | 0.02 | 0.03 | 0.03 | 0.01 | 0.01 | 0.04 | 0.01 |
|  |  | (0.16) | (0.23) | (0.20) | (0.07) | (0.11) | (0.23) | (0.06) |
|  | ΣBPDs | 2.11 | 3.29 | 2.87 | 0.78 | 1.37 | 3.78 | 1.11 |
|  |  | (16.27) | (24.26) | (18.50) | (7.49) | (10.00) | (21.48) | (6.02) |
| Cereals  Mean (P97.5) | | 115.7 | 24.3 | 7.0 | 3.4 | 3.5 | 14.2 | 30.9 |
|  |  | (760.3) | (158.2) | (45.0) | (24.7) | (23.5) | (96.4) | (196.0) |

Table S4 The mean (P97.5) values of EDI for BP and BPDs using the MC approach for three types of cereals and for each age group

| EDI (ng/kg/day) | BPDs | Age group (years old) | | | | | | |
| --- | --- | --- | --- | --- | --- | --- | --- | --- |
|  |  | **0-3** | **3-6** | **6-12** | **12-16** | **16-18** | **19-65** | **>65** |
| Rice flour,  Mean  (P97.5) | BP | 78.56 | 10.71 | 0.35 | 0.74 | 0.15 | 2.19 | 4.82 |
|  |  | (476.72) | (72.47) | (2.44) | (4.72) | (1.09) | (15.80) | (32.76) |
|  | 4-MBP | 11.93 | 1.28 | 0.07 | 0.09 | 0.02 | 0.31 | 0.62 |
|  |  | (53.89) | (7.50) | (0.21) | (0.38) | (0.12) | (1.52) | (4.00) |
|  | BP-3 | 1.03 | 0.12 | 0.00 | 0.01 | 0.00 | 0.03 | 0.06 |
|  |  | (5.40) | (0.79) | (0.02) | (0.05) | (0.01) | (0.16) | (0.45) |
|  | ΣBPDs | 91.51 | 12.12 | 0.42 | 0.84 | 0.18 | 2.53 | 5.50 |
|  |  | (536.01) | (80.76) | (2.68) | (5.16) | (1.22) | (17.47) | (37.20) |
| Oatmeal,  Mean  (P97.5) | BP | 49.16 | 6.92 | 3.40 | 1.45 | 1.69 | 7.40 | 20.16 |
|  |  | (270.23) | (54.31) | (31.13) | (10.29) | (11.17) | (51.41) | (130.51) |
|  | 4-MBP | 15.29 | 1.79 | 0.87 | 0.35 | 0.29 | 2.06 | 5.16 |
|  |  | (60.84) | (21.90) | (5.43) | (2.86) | (2.36) | (11.92) | (25.47) |
|  | BP-3 | 0.57 | 0.06 | 0.03 | 0.01 | 0.02 | 0.07 | 0.18 |
|  |  | (2.77) | (0.40) | (0.23) | (0.10) | (0.10) | (0.40) | (1.11) |
|  | ΣBPDs | 65.02 | 8.78 | 4.30 | 1.81 | 2.00 | 9.52 | 25.51 |
|  |  | (333.84) | (76.60) | (36.79) | (13.24) | (13.64) | (63.74) | (157.09) |
| Corn flakes,  Mean  (P97.5) | BP | 28.49 | 3.01 | 1.64 | 1.01 | 1.76 | 1.61 | 1.07 |
|  |  | (140.51) | (23.14) | (8.76) | (7.33) | (11.59) | (11.35) | (5.50) |
|  | 4-MBP | 2.62 | 0.13 | 0.07 | 0.05 | 0.07 | 0.09 | 0.04 |
|  |  | (5.43) | (0.94) | (0.50) | (0.33) | (0.49) | (0.57) | (0.27) |
|  | BP-3 | 0.27 | 0.05 | 0.03 | 0.01 | 0.02 | 0.02 | 0.01 |
|  |  | (1.34) | (0.28) | (0.13) | (0.10) | (0.11) | (0.13) | (0.08) |
|  | ΣBPDs | 31.38 | 3.19 | 1.74 | 1.07 | 1.85 | 1.73 | 1.12 |
|  |  | (147.28) | (24.36) | (9.38) | (7.77) | (12.20) | (12.05) | (5.85) |
| Cereals  Mean (P97.5) | | 187.9 | 24.1 | 6.5 | 3.7 | 4.0 | 13.8 | 32.1 |
|  |  | (1017.1) | (181.7) | (48.8) | (26.2) | (27.1) | (93.3) | (200.2) |

Table S5 The mean (P97.5) HQs for BP and BPDs using the MCMC method by cereal type and age group

| HQ | BPDs | Age group (years old) | | | | | | |
| --- | --- | --- | --- | --- | --- | --- | --- | --- |
|  |  | **0-3** | **3-6** | **6-12** | **12-16** | **16-18** | **19-65** | **>65** |
| Rice flour,  Mean (P97.5) | BP | 2.5E-03 | 3.4E-04 | 1.2E-05 | 2.5E-05 | 5.4E-06 | 7.0E-05 | 1.5E-04 |
|  |  | (1.5E-02) | (2.5E-03) | (8.3E-05) | (1.6E-04) | (3.5E-05) | (5.0E-04) | (1.1E-03) |
|  | 4-MBP | 1.2E-04 | 1.6E-05 | 5.8E-07 | 1.2E-06 | 2.6E-07 | 3.3E-06 | 7.3E-06 |
|  |  | (7.3E-04) | (1.2E-04) | (3.9E-06) | (7.7E-06) | (1.6E-06) | (2.3E-05) | (5.0E-05) |
|  | BP-3 | 7.4E-06 | 1.0E-06 | 3.7E-08 | 7.7E-08 | 1.6E-08 | 2.1E-07 | 4.7E-07 |
|  |  | (4.4E-05) | (7.9E-06) | (2.5E-07) | (4.8E-07) | (1.1E-07) | (1.5E-06) | (3.1E-06) |
|  | ΣBPDs | 2.6E-03 | 3.6E-04 | 1.3E-05 | 2.7E-05 | 5.7E-06 | 7.4E-05 | 1.6E-04 |
|  |  | (1.6E-02) | (2.6E-03) | (8.7E-05) | (1.7E-04) | (3.7E-05) | (5.2E-04) | (1.1E-03) |
| Oatmeal,  Mean (P97.5) | BP | 9.8E-04 | 1.1E-04 | 5.4E-05 | 2.3E-04 | 3.4E-05 | 3.5E-04 | 5.2E-04 |
|  |  | (7.5E-03) | (6.7E-04) | (3.5E-04) | (1.7E-03) | (1.1E-04) | (1.0E-03) | (1.5E-03) |
|  | 4-MBP | 1.7E-04 | 1.9E-05 | 9.7E-06 | 4.0E-05 | 5.8E-06 | 6.1E-05 | 9.5E-05 |
|  |  | (1.3E-03) | (1.2E-04) | (6.3E-05) | (2.9E-04) | (1.8E-05) | (1.8E-04) | (2.7E-04) |
|  | BP-3 | 2.6E-06 | 3.0E-07 | 1.5E-07 | 6.1E-07 | 9.3E-08 | 9.7E-07 | 1.4E-06 |
|  |  | (2.1E-05) | (1.8E-06) | (9.7E-07) | (4.0E-06) | (3.4E-07) | (3.3E-06) | (4.4E-06) |
|  | ΣBPDs | 1.2E-03 | 1.2E-04 | 6.4E-05 | 2.7E-04 | 4.0E-05 | 4.1E-04 | 6.2E-04 |
|  |  | (8.9E-03) | (7.9E-04) | (4.1E-04) | (2.0E-03) | (1.3E-04) | (1.2E-03) | (1.8E-03) |
| Corn flakes,  Mean (P97.5) | BP | 6.7E-05 | 1.0E-04 | 9.1E-05 | 2.5E-05 | 4.4E-05 | 1.2E-04 | 3.5E-05 |
|  |  | (5.2E-04) | (7.7E-04) | (5.9E-04) | (2.4E-04) | (3.2E-04) | (6.8E-04) | (1.9E-04) |
|  | 4-MBP | 2.7E-06 | 4.0E-06 | 3.5E-06 | 9.7E-07 | 1.7E-06 | 4.8E-06 | 1.3E-06 |
|  |  | (1.9E-05) | (3.0E-05) | (2.3E-05) | (9.2E-06) | (1.2E-05) | (2.7E-05) | (8.2E-06) |
|  | BP-3 | 2.1E-07 | 3.3E-07 | 2.8E-07 | 8.0E-08 | 1.4E-07 | 3.8E-07 | 1.0E-07 |
|  |  | (1.6E-06) | (2.3E-06) | (2.0E-06) | (7.2E-07) | (1.1E-06) | (2.3E-06) | (6.2E-07) |
|  | ΣBPDs | 7.0E-05 | 1.1E-04 | 9.5E-05 | 2.6E-05 | 4.5E-05 | 1.3E-04 | 3.7E-05 |
|  |  | (5.4E-04) | (8.0E-04) | (6.1E-04) | (2.5E-04) | (3.3E-04) | (7.1E-04) | (2.0E-04) |
| Cereals  Mean (P97.5) | | 3.8E-03 | 5.9E-04 | 1.7E-04 | 3.2E-04 | 9.1E-05 | 6.1E-04 | 8.2E-04 |
|  |  | (2.5E-02) | (4.2E-03) | (1.1E-03) | (2.4E-03) | (4.9E-04) | (2.4E-03) | (3.1E-03) |

Table S6 The mean (P97.5) HQs for BP and BPDs using the MC method by cereal type and age group

| HQ | BPD | Age group (years old) | | | | | | | | | | | | |
| --- | --- | --- | --- | --- | --- | --- | --- | --- | --- | --- | --- | --- | --- | --- |
|  |  | **0-3** | **3-6** | | **6-12** | | **12-16** | | | **16-18** | | **19-65** | | **>65** |
| Rice flour,  Mean (P97.5) | BP | 2.6E-03 | 3.6E-04 | | 1.2E-05 | | 2.5E-05 | | | 5.1E-06 | | 7.3E-05 | | 1.6E-04 |
|  |  | (1.6E-02) | (2.4E-03) | | (8.1E-05) | | (1.6E-04) | | | (3.6E-05) | | (5.3E-04) | | (1.1E-03) |
|  | 4-MBP | 4.0E-04 | 4.3E-05 | | 2.3E-06 | | 2.9E-06 | | | 8.1E-07 | | 1.0E-05 | | 2.1E-05 |
|  |  | (1.8E-03) | (2.5E-04) | | (7.1E-06) | | (1.3E-05) | | | (4.1E-06) | | (5.1E-05) | | (1.3E-04) |
|  | BP-3 | 1.0E-05 | 1.2E-06 | | 4.9E-08 | | 1.1E-07 | | | 2.0E-08 | | 3.0E-07 | | 5.6E-07 |
|  |  | (5.4E-05) | (7.9E-06) | | (2.4E-07) | | (5.1E-07) | | | (1.3E-07) | | (1.6E-06) | | (4.5E-06) |
|  | ΣBPDs | 3.0E-03 | 4.0E-04 | | 1.4E-05 | | 2.8E-05 | | | 6.0E-06 | | 8.4E-05 | | 1.8E-04 |
|  |  | (1.8E-02) | (2.7E-03) | | (8.9E-05) | | (1.7E-04) | | | (4.0E-05) | | (5.8E-04) | | (1.2E-03) |
| Oatmeal,  Mean (P97.5) | BP | 8.9E-03 | 1.6E-03 | | 4.3E-04 | | 2.1E-04 | | | 2.6E-04 | | 7.9E-04 | | 1.2E-03 |
|  |  | (5.1E-03) | (9.2E-04) | | (4.5E-04) | | (2.1E-04) | | | (2.3E-04) | | (8.9E-04) | | (2.7E-03) |
|  | 4-MBP | 8.7E-03 | 7.3E-04 | | 2.5E-04 | | 7.9E-05 | | | 4.0E-05 | | 6.1E-04 | | 1.7E-03 |
|  |  | (8.0E-04) | (1.4E-04) | | (8.2E-05) | | (3.2E-05) | | | (3.4E-05) | | (1.8E-04) | | (4.5E-04) |
|  | BP-3 | 6.6E-05 | 5.4E-06 | | 1.4E-06 | | 5.5E-07 | | | 1.3E-06 | | 2.5E-06 | | 5.4E-06 |
|  |  | (1.3E-05) | (1.8E-06) | | (1.0E-06) | | (5.0E-07) | | | (5.5E-07) | | (2.3E-06) | | (7.2E-06) |
|  | ΣBPDs | 1.8E-02 | 2.3E-03 | | 6.8E-04 | | 2.9E-04 | | | 3.0E-04 | | 1.4E-03 | | 2.9E-03 |
|  |  | (5.9E-03) | (1.1E-03) | | (5.3E-04) | | (2.4E-04) | | | (2.6E-04) | | (1.1E-03) | | (3.2E-03) |
| Corn flakes,  Mean (P97.5) | BP | 9.5E-04 | 1.0E-04 | | 5.5E-05 | | 3.4E-05 | | | 5.9E-05 | | 5.4E-05 | | 3.6E-05 |
|  |  | (4.7E-03) | (7.7E-04) | | (4.3E-04) | | (2.4E-04) | | | (3.9E-04) | | (3.8E-04) | | (1.8E-04) |
|  | 4-MBP | 8.7E-05 | 4.3E-06 | | 2.3E-06 | | 1.6E-06 | | | 2.4E-06 | | 3.0E-06 | | 1.3E-06 |
|  |  | (1.8E-04) | (3.1E-05) | | (1.7E-05) | | (1.1E-05) | | | (1.6E-05) | | (1.9E-05) | | (9.0E-06) |
|  | BP-3 | 2.7E-06 | 5.0E-07 | | 3.0E-07 | | 1.5E-07 | | | 1.7E-07 | | 2.4E-07 | | 1.4E-07 |
|  |  | (1.3E-05) | (2.8E-06) | | (1.3E-06) | | (9.8E-07) | | | (1.1E-06) | | (1.3E-06) | | (7.9E-07) |
|  | ΣBPDs | 1.0E-03 | 1.1E-04 | | 5.7E-05 | | 3.5E-05 | | | 6.1E-05 | | 5.7E-05 | | 3.7E-05 |
|  |  | (4.9E-03) | (8.1E-04) | | (4.4E-04) | | (2.6E-04) | | | (4.0E-04) | | (4.0E-04) | | (1.9E-04) |
| Cereals  Mean (P97.5) | | 2.2E-02 | | 2.8E-03 | | 7.5E-04 | | 3.5E-04 | 3.7E-04 | | 1.5E-03 | | 3.1E-03 | |
|  |  | (2.9E-02) | | (4.5E-03) | | (1.1E-03) | | (6.7E-04) | (7.0E-04) | | (2.0E-03) | | (4.6E-03) | |


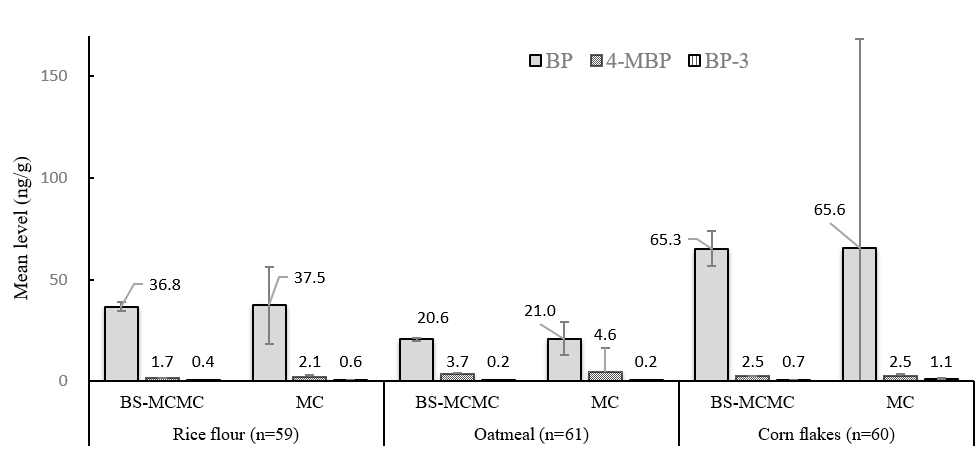


Figure S1. Comparison of means (SDs) of BP, 4-MBP, and BP-3 distributions using the MCMC and MC methods for rice flour, oatmeal, and corn flakes.

Figure S2. The P97.5 values of the dietary intake estimated using the MC

approach by cereal type and age group

Figure S3. The P97.5 values of the HIs using MC simulation by cereal type

and age group

Figure S4. Sensitivity analysis of each parameter’s contribution to the BPDs HIs using the MC method for older adults aged >65 years
